# Uncommon Pathogens in Common Presentations: Genetic Profiling and Virulence Determinants of *Vibrio alginolyticus* Isolated from a Case of External Otitis

**DOI:** 10.3390/idr17050114

**Published:** 2025-09-12

**Authors:** Radu Ovidiu Togănel, Razvan Lucian Coșeriu, Anca Delia Mare, Camelia Vintilă, Ioan-Ovidiu Sîrbu, Aimée Rodica Chis, Cristina Elena Gîrbovan, Adrian Man

**Affiliations:** 1 Microbiology Department, George Emil Palade University of Medicine, Pharmacy, Science, and Technology of Targu Mures, 540142 Targu Mures, Romania; radu.toganel@umfst.ro (R.O.T.); anca.mare@umfst.ro (A.D.M.); adrian.man@umfst.ro (A.M.); 2 Doctoral School of Medicine and Pharmacy, George Emil Palade University of Medicine, Pharmacy, Science, and Technology of Targu Mures, 540142 Targu Mures, Romania; vintilacamelia@yahoo.com; 3 Biochemistry Department, “Victor Babes” University of Medicine and Pharmacy Timisoara, 300041 Timisoara, Romania; ovidiu.sirbu@umft.ro (I.-O.S.); chis.aimee@umft.ro (A.R.C.); 4 Center for Complex Networks Science, “Victor Babes” University of Medicine and Pharmacy Timisoara, 300041 Timisoara, Romania; 5 Department of Infectious Diseases, George Emil Palade University of Medicine, Pharmacy, Science, and Technology of Targu Mures, 540142 Targu Mures, Romania; elena.girbovan@umfst.ro

**Keywords:** *Vibrio alginolyticus*, otitis externa, whole genome sequencing, virulence

## Abstract

**Backgrunod/Objectives**: Routine identification of common bacterial pathogens is typically efficient, utilizing standardized, cost-effective methods. However, the diagnostic process becomes significantly more complex when dealing with rare or unexpected microorganisms, especially as they can be considered colonizers in many cases. **Methods**: This study presents diagnostic details of an uncommon pathogen, *Vibrio alginolyticus*, isolated from auricular discharge in a patient with non-Hodgkin lymphoma diagnosed with persistent otitis externa and explores its identification through both conventional and modern laboratory approaches. Sequential ear discharge cultures resulted in phenotypically similar but genomically different *Vibrio alginolyticus* isolates. We complemented classical methods like conventional culture (on Columbia agar and CLED agar), Vitek2 Compact identification, and EUCAST disk diffusion antimicrobial susceptibility testing (following the EUCAST version 12.0 guidelines) with MALDI-TOF mass spectrometry and Illumina/Nanopore whole genome sequencing. Comparative analysis of the genomes was performed with the PeGAS pipeline, Unicycler, and 1928Diagnostics SNP analysis. **Results**: The Vitek2 analysis identified both isolates as *V. alginolyticus* with 99% confidence, and this was supported by the MALDI-TOF MS results. The first isolate (A) was fully susceptible to the antibiotics tested, while the second (B) showed resistance to ciprofloxacin. Whole genome sequencing revealed 99.23% and 98.60% nucleotide identity to the *V. alginolyticus* reference genome for isolates A and B, respectively, with a 99.8% match between them. Isolate B acquired a *gyrA* (c.1870C>T) mutation that correlates with the ciprofloxacin resistance (MIC > 0.5 mg/L). Both genomes carry *hlyA* (hemolysin), *toxR* (cholera toxin regulator), genes involved in biofilm formation (*rpoN*, *relA*, *spoT*, *opp*), *luxS* (motility), *proA*, *vacB* (virulence factors), and *tet(34)* (oxytetracycline resistance). A core genome SNP distance of <100 indicates clonal relatedness. Our integrated (phenotypic and genomic) diagnostic approach confirmed *V. alginolyticus* and documented host resistance evolution, with a virulence repertoire that could explain the clinical evolution. **Conclusions**: This case highlights the utility of molecular methods in confirming species identity, detecting resistance markers, characterizing virulence determinants, and differentiating a pathogen from a colonizer, supporting targeted clinical management.

## 1. Introduction

Otitis externa, the inflammation of the ear canal, is one of the most common afflictions in the ENT (ear, nose, and throat) departments [1,2]. Infectious otitis externa is caused by both bacteria and fungi, with frequent bacterial pathogens being *Pseudomonas aeruginosa*, *Staphylococcus aureus*, and *Streptococcus pyogenes* [1]. Risk factors include trauma of the ear canal, dermatological conditions, modifications of local immunity, including diabetes [3], and water in the ear canal (especially while swimming) [4,5]. Although *Pseudomonas aeruginosa*, *Staphylococcus aureus*, and *Streptococcus pyogenes* account for the majority of cases, a growing body of evidence implicates vibrios, including *Vibrio alginolyticus* [1].

*V. alginolyticus* is a halophilic Gram-negative vibrion usually found in marine environments, especially in warm coastal waters [1,6,7,8]. It is a significant pathogen in aquaculture, affecting fish and shellfish, with an impact on economics [9]. Moreover, it is reported to be a human pathogen, causing wound infections, eye infections, and ear infections, as well as gastrointestinal tract infections and systemic infections [1,6,7,8,10,11,12]. Identification of *V. alginolyticus* is challenging because vibrios may be dismissed as colonizers or misidentified by automated systems.

Although sporadic reports of *V. alginolyticus* otitis externa exist in the United States and parts of Asia, the evidence from European countries remains scarce, with only isolated cases described to date. Slifka et al. (2017) conducted an epidemiological study in the US and reported 437 cases between 1988 and 2012 [6]. Citil et al. (2015) reported four cases in Turkey [8], while Reilly et al. (2011) suggested that these cases are rare in Europe [7]. This report contributes to the scarce literature on *V. alginolyticus* by documenting a case of otitis externa in Romania, thereby extending the geographical range of reported infections in Europe. Given the uncommon involvement of this species in ear infections and the diagnostic challenges encountered both clinically and paraclinically, confirmation may require advanced methodologies. Here, we present a comprehensive genomic and phenotypic characterization of *V. alginolyticus* isolated from an otitis externa case in Romania, employing conventional diagnostic techniques (culture and antimicrobial susceptibility testing) in conjunction with whole genome sequencing (WGS). The findings underscore the organism’s potential for rapid antimicrobial resistance evolution and its clinical relevance in non-endemic settings.

## 2. Materials and Methods

A 43-year-old female patient diagnosed with non-Hodgkin lymphoma in 2014, with a relapse in 2021, was admitted to the Infectious Disease ward of the Mureș Clinical County Hospital Mureș (Targu Mures, Romania) with symptoms of external otitis and with no other relevant medical history. Two ear discharge samples (collected 16 August 2022, and 12 September 2022) were cultured on standard media (CLED agar and Columbia agar) and were identified/processed on VITEK^®^-2 COMPACT (bioMérieux, Marcy l’Etoile, France) followed by antibiotic susceptibility testing (AST) performed using the Kirby–Bauer disk diffusion method interpreted with EUCAST v12.0 (The European Committee on Antimicrobial Susceptibility Testing) breakpoints, concluding the routine diagnosis of the case, given the limited infrastructure of the clinical laboratory. Clinical data reporting and bacterial isolate analysis were performed with approval from the Hospital Ethics Committee (No. 13699, approved on 9 November 2022), and further analyses were conducted in collaborating partner university research laboratories to validate the diagnosis and confirm the pathogen’s role in disease etiology.

Species-level confirmation was performed using Matrix-Assisted Laser Desorption/Ionization Time-of-Flight Mass Spectrometry on a MALDI Biotyper^®^ (Version 2.0, Bruker, Bremen, Germany).

Genomic bacterial isolate DNA was purified with the Quick-DNATM Fungal/Bacterial Miniprep Kit (Zymo Research, Irvine, CA, USA) according to the instructions of the producer. The DNA quantification was performed with 1 µL of DNA eluate using the D30 BioPhotometer (Eppendorf, Hamburg, Germany).

The whole genome sequencing (WGS) was performed by two methods, using Illumina Next-Generation Paired-End Sequencing and Oxford Nanopore Single-end sequencing, respectively.

Genomic libraries were prepared using the Nextera DNA Prep kit (Illumina) following the manufacturer’s protocol. Paired-end sequencing (2 × 150 bp) was performed on the Illumina MiSeq platform using the MiSeq Reagent Kit (600-cycle format) (v3, Illumina, San Diego, CA, USA).

For Nanopore sequencing, we employed the Rapid Barcoding Kit 24 (SQK-RBK114.24, Oxford Nanopore Technologies (ONT), Oxford, UK) following the manufacturer’s protocol. The library was quantified using the dsDNA HS Assay Kit (Thermo Fisher, Waltham, MA, USA) on a Qubit 2.0 Fluorometer (Thermo Fisher Scientific, Waltham, MA, USA) and further loaded on a primed R10.4.1 flow cell (ONT, Oxford, UK). The sequencing experiment was performed on a MinION Mk1B instrument (ONT, Oxford, UK). MinKNOW software v.24.11.10 was used for raw data processing and Dorado v7.6.8 (Dorado Software, El Dorado Hills, CA, USA) was used for basecalling, adaptor trimming, and demultiplexing.

Quality control (FastQC), hybrid assembly (Unicycler), annotation (Prokka), and resistome–virulome screening (Abricate, VFDB, ResFinder) were executed in the PeGAS pipeline [13]. Average nucleotide identity (ANI) was calculated with fastANI. Single-Nucleotide Polymorphism (SNP analysis) was used for both reads, provided by a commercially available platform (1928 Diagnostics). The NCBI Basic Local Alignment Search Tool (BLAST) (National Center for Biotechnology Information, Bethesda, MD, USA) was used to align the sequenced genomes with the *Vibrio alginolyticus* reference genome, performed on the official website (http://www.ncbi.nlm.nih.gov/BLAST/, accessed on 28 June 2023).

## 3. Results

Both isolates were identified as *Vibrio alginolyticus* (99% confidence), both by MALDI-TOF MS analysis and by Vitek2 Compact. Isolate A was susceptible to all tested antibiotics, including ceftazidime, cefotaxime, piperacillin–tazobactam, erythromycin, trimethoprim–sulfamethoxazole, ciprofloxacin, and levofloxacin. Isolate B exhibited ciprofloxacin resistance (12mm inhibition zone) while remaining susceptible to levofloxacin, β-lactams, and trimethoprim–sulfamethoxazole. Whole genome sequencing revealed 99.23% and 98.60% similarity with the reference genome for isolates A and B, respectively, with a 99.8% nucleotide match between them.

Given the ciprofloxacin resistance, an SNP analysis was performed against the *V. alginolyticus* reference genome E110 (GCF_023650915.1), showing mutations in the *gyrA/gyrB* and *parE* genes (Table 1).

The PeGAS report showed 43% and 44% GC content in isolates A and B, respectively. The antibiotic resistance genes detected on both assemblies were *blaCARB-42* (carbenicillin-hydrolyzing class A beta-lactamase CARB-42), with 99.77% identity and 100% coverage, *tet(34)* (oxytetracycline resistance phosphoribosyltransferase domain-containing protein Tet(34)), with 83.01% identity and 100% coverage for isolate A and 99.78% for isolate B, and *tet(35)* (tetracycline efflux Na+/H+ antiporter family transporter Tet(35)), with 83.71% identity, 100% coverage for isolate A, and 83.64% identity, 99.94% coverage for isolate B. We identified 11 virulence factors, which are presented in Table 2.

For isolate A, we identified the presence of the following colonization genes: hemolysin *hlyA*, *toxR*, and *collagenase*. We also found *rpoN*, *relA*, *spoT*, and *opp*, which are associated with biofilm formation. In addition, we detected *luxS*, a flagellum-encoding gene. The virulence factor *proA* and the resistance gene *tet(34)* were also present. Analysis of the isolate B identified colonization genes hemolysin *hlyA*, *toxR*, *collagenase*, and *ompW*. We also found *rpoN*, *relA*, *spoT*, and *opp*, which are involved in biofilm formation. Furthermore, the flagellum gene *luxS*, the virulence gene *vacB,* and the resistance gene *tet(34)* were identified. Details about the NCBI BLAST results are presented in Table 3.

## 4. Discussion

This report underscores three clinically relevant insights: (i) vibrios can establish chronic infection in immunocompromised hosts; (ii) within-host fluoroquinolone resistance can quickly emerge over weeks through QRDR mutation; and (iii) *V. alginolyticus* possesses an extensive virulence arsenal, including T3SS and hemolysins, which may drive tissue invasion and impair healing. Early incorporation of MALDI-TOF MS and WGS accelerated definitive diagnosis and guided the switch to topical levofloxacin, resulting in resolution.

*Vibrio* species have long been recognized as opportunistic human pathogens, most probably due to the rise in global marine water temperature and increased coastal recreational tourism. US data show a consistent increase in *Vibrio* infections, approximately 17 being associated early with otitis externa [6]. Our findings outline the need to broaden the differential diagnosis of chronic “swimmer’s ear,” especially in immunocompromised individuals.

Rapid, comprehensive species confirmation demands a multi-layered diagnostic strategy; MALDI-TOF rapidly validated the Vitek2 results, while whole genome (Illumina/Nanopore) sequencing further characterized the species and produced resistome/virulome data. Recent studies showed that WGS can be routinely used to identify *Vibrio* species harboring antibiotic resistance loci [14].

Both sequential isolates carry an intact 11-gene type III secretion system (T3SS), known to promote tissue invasion and biofilm maturation [15]. Additional determinants like *tlh* (thermolysin), *hlyA* (hemolysin), quorum-sensing regulator *luxS*, and master regulator *toxR* were identified as possible causes of persistence of infection and incomplete response to standard ciprofloxacin–dexamethasone therapy. Characterizing a complete virulence tableau is instrumental for therapeutic decisions like prolonged topical therapy, debridement, and strict post-treatment surveillance.

### 4.1. Antibiotic Resistance Genes

The *gyrA* and *gyrB* genes encode the A and B subunits of the DNA gyrase in bacteria, while quinolones inhibit the activity of the DNA gyrase. Mutations in the *gyrA/gyrB* genes were previously described by several authors as being involved in quinolone resistance among different bacteria (*Pseudomonas aeruginosa*, *Escherichia coli*, *Klebsiella pneumoniae*, *Staphylococcus aureus*, *Mycobacterium tuberculosis*). However, the most commonly codons related to quinolones are 83 and 87, whereas our finding was a strikingly rapid acquisition (over just four weeks) of punctiform mutation c.1870C>T, which corresponds to codon 623, situated near the C-terminal region of the *gyrA* gene, not changing the codon translation and not in the Quinolone Resistance-Determining Region (QRDR) [16,17,18,19,20]. The *parE* gene encodes one of the two subunits of topoisomerase IV, also related to ciprofloxacin resistance. A mutation in the QRDR of the *parE* is described among codons 445-464 [21] but is not present in our case. Nevertheless, the second isolate phenotypically expressed fluoroquinolone resistance. This can be explained by plasmid-mediated quinolone resistance (PMQR) due to *qnrA/B/S* plasmid-mediated expression or by the overexpression of efflux pumps, such as *norM*, *vcmA*, or *vcaM*, both of which have been described in *Vibrio* spp. [22,23]. Thus, while the SNP was detected during comparative analysis, the resistance phenotype is more plausibly attributable to additional mechanisms that were not fully captured in our genomic analysis.

The *qnr* genes encode pentapeptide repeat proteins that bind to DNA gyrase and topoisomerase IV, protecting them from inhibition by fluoroquinolones [24]. *Qnr*-producing strains often have low-level resistance, which may be enough to push ciprofloxacin above the resistance breakpoint (MIC > 0.25 mg/L) [25], but not levofloxacin or norfloxacin [26], especially if associated with the presence of aac(6′)-Ib-cr aminoglycoside acetyltransferase [27]. Such low-level resistance can act synergistically with efflux pump overexpression to produce clinically relevant phenotypes. While our genomic pipeline did not identify a *qnr* determinant with high confidence, the phenotypic profile of isolate B (ciprofloxacin resistance with retained levofloxacin susceptibility) is consistent with PMQR involvement.

Both *Vibrio alginolyticus* strains showed the presence of the *blaCARB-42*, *tet(34)*, and *tet(35)* genes, in line with previous reports on *V. alginolyticus* antibiotic resistance [28]. The *blaCARB-42* gene is relevant for the ampicillin, amoxicillin, and piperacillin resistance, *tet(34)* is involved in tetracycline resistance, and *tet(35)* is known to produce doxycycline resistance among *Vibrio* species. These findings were previously described by other authors during genomic analysis of *Vibrio alginolyticus*, suggesting a common characteristic of the species [29,30,31,32,33,34].

The ciprofloxacin resistance of the second isolate might have a clinical impact, given that ciprofloxacin is a common antibiotic used in the topical treatment of bacterial otitis externa [3].

### 4.2. Virulence Factors

The *tlh* gene (encoding the thermolabile hemolysin—a major virulence factor of *Vibrio* spp.) was present in both our isolates [35]. The hemolysin is mentioned to be involved in pathogenicity by lysing the cell wall of erythrocytes and other cells, such as neutrophils, leading to lysis. Then, bacteria transfer the compounds (iron, proteins) through the cellular membrane by various receptors [36,37]. The presence of the *tlh* gene in both *Vibrio* isolates suggests a potential contribution of thermolabile hemolysin to the pathogenesis of external otitis by promoting local tissue damage, impairing innate immune responses, and facilitating nutrient acquisition, thus enhancing bacterial survival in the external ear canal.

A type III secretion system (T3SS) regulatory protein encoded by the *tyeA* gene was found in the genome of our isolates, suggesting a role in chronic tissue colonization and impaired healing, particularly in the immunocompromised host. The TyeA protein is described by Zhao et al. (2011) to be located in the membrane of the cell, yet its role still remains ambiguous [35]. Zhou et al. (2020) and Zhang et al. (2022) suggest that it has an involvement in protein expression regulation [38,39]. Multiple other genes linked to the T3SS (*vcrH*, *vopB*, *vopD*, *vopR*, *vscF*, *vscI*, *vscN*, *vscR*, *vscS*) were present. T3SS is present in many Gram-negative species (*Salmonella* spp., *Shigella* spp., *Escherichia coli*, *Pseudomonas aeruginosa*, *Vibrio* spp.), with a major role in pathogenicity [40]. According to the model proposed by Abrusci et al. (2014), there are two types of T3SS—flagellar and non-flagellar. The architecture of the non-flagellar system consists of a multiple-ring configuration placed on both sides of the cellular membrane, powered by an ATPase complex and connected to a needle through which virulence proteins are injected into the host, with the help of the chaperones [40,41]. The virulence mediated by the T3SS effectors was mentioned by Kodama et al. (2015) in terms of enterotoxicity for *V. parahaemolyticus*, with demonstrated action on the actin cytoskeleton [42]. However, Goldufsky et al. (2015) demonstrated the role of T3SS in inhibiting the healing of the diabetic ulcer in wounds infected with *Pseudomonas aeruginosa*, denoting that, due to decreased local immune response, the secretory system of the bacteria may alter the healing process [43]. Given the fact that the patient from whom we isolated the *V. alginolyticus* strains was diagnosed with non-Hodgkin lymphoma and treated with cytostatic drugs, the presence of T3SS might be a factor related to the treatment failure of the ear infection, although it was not specifically demonstrated. From a therapeutic perspective, T3SS-mediated biofilm formation and persistence can reduce the efficacy of antibiotics and delay clinical resolution. This highlights the potential need for prolonged or combination therapy and close post-treatment monitoring in cases of *V. alginolyticus* otitis externa.

HlyA (α-hemolysin) activity was described over 30 years ago by many authors in *Escherichia coli*, *Vibrio cholerae*, and other Gram-negative bacteria. It is part of the Type 1 Secretion System (T1SS) [44]. The 110kDa protein is encoded by the *hlyA* gene and is part of the *hly* region of the chromosomal DNA of bacteria, and its activity is strongly related to calcium bonding, as proven by Boehm et al. (1990), a crucial element for attachment of the protein to the erythrocyte membrane, inducing hemolysis and being a major virulence factor. The lytic effect is not limited to red blood cells; epithelial and endothelial cells can also be targeted [45,46,47,48,49,50].

The *toxR* gene was initially described as a regulatory gene for the *V. cholerae* toxin operon, but later on, it was sequenced in other *Vibrio* species, proving that it is consistent among the genus [51,52]. The ToxR regulon is a critical part of a much larger regulation cascade, and it works in tandem with another protein, ToxS [53]. As a mechanism of action, the ToxS-ToxR pair binds to the DNA and activates transcription of many other virulence genes [54]. Kazi et al. (2016) suggested an experimental model that demonstrated the impact of the ToxR protein on intestinal colonization for *Vibrio cholerae* [55]. ToxR enables *Vibrio* to sense and adapt to specific environmental conditions, including those of the ear canal, which is moist, slightly acidic, nutrient-limited, and has important local defense mechanisms.

The outer membrane of Gram-negative bacteria plays a major role in bacterial survival in different environments, and the most important components are the membrane proteins situated on the outer membrane of the cells [56]. OmpW is one of the proteins detected in the analyzed *V. alginolyticus* strains, with a demonstrated role in protection against the host’s immune system, specifically phagocytosis [56,57]. Studies on other Gram-negative bacteria, such as *Acinetobacter baumannii*, have demonstrated the involvement of this protein in iron uptake, with increased *ompW* gene expression. However, gene expression may be influenced by antibiotic exposure, and the protein’s structure could be altered by drugs like colistin, potentially affecting the bacterial virulence [58,59,60]. Other OMPs are relevant for the virulence of *Vibrio* spp., such as OmpU, OmpK, and OmpW, playing a central role in pathogenesis by mediating host cell adhesion, nutrient uptake, immune evasion, and antimicrobial resistance [61]. OMPs are attractive vaccine candidates due to their surface exposure, high immunogenicity, conservation among strains, and involvement in critical virulence functions [62].

Multiple reports of RpoN (RNA polymerase sigma factor N), an alternative sigma factor, were found in various *Vibrio* species and in other pathogens. The activity of the protein relates to DNA transcription, influencing genes involved in processes, including biofilm formation, cell adhesion, motility, and structural assembly [63]. RpoN was reported to control the expression of over 130 genes responsible for encoding the type VI secretion system (T6SS) of diverse bacteria [63]. RpoN is known to have a role in adhesion and biofilm dynamics [64]. Sheng et al. (2012) determined that the T6SS substrate Hcp1 was controlled, among others (quorum-sensing system and binding protein VasH), by the RpoN sigma factor in *V. alginolyticus* [65]. Zhang et al. (2021) experimentally noted that, in *V. alginolyticus* HN08155, biofilm formation occurs at low cell density and detaches at high cell density, and the sigma factor RpoN is not required for biofilm formation; however, it plays a role in biofilm detachment [66]. In the context of external otitis, the RpoN sigma factor in *Vibrio* species likely contributes to pathogenesis by coordinating virulence programs, such as T6SS-mediated competition, adhesion, and biofilm modulation, leading to a stable colonization of the auditory canal and the transition to invasive infection through biofilm dispersal and epithelial interaction.

*RelA* and *spoT* genes found in both our sequences are accessory genes, highly dynamic in evolution and dependent on the environment, which encode two proteins, RelA and SpoT, respectively, part of a system called RelA/SpoT Homologue (RSH). The system regulates the activity of the ppGpp (guanosine 5′-diphosphate, 3′-diphosphate) alarmone, which is secreted in response to environmental stress by the bacterial cell. The ppGpp system produces a stringent response to amino acid starvation, leading to SpoT synthesis, according to Yin et al. (2021). Furthermore, Yin et al. (2022) related that the *spoT* deficiency in *Vibrio alginolyticus* may lead to morphological abnormalities (loss of flagella), and, consequently, delays in biofilm formation, probably due to the flagellar aggregation [67,68,69]. The ppGpp-mediated stringent response allows *Vibrio* to adjust its metabolic activity and virulence expression for survival and persistence. By modulating growth, biofilm formation, and virulence in response to stress, this system likely contributes to the persistence and pathogenesis of *Vibrio*-related otitis externa [68].

In *Vibrio alginolyticus*, the *opp* gene cluster consists of five genes: *oppA*, *oppB*, *oppC*, *oppD*, and *oppF*. Generally, it encodes the oligopeptide permease system in both Gram-positive bacteria and Gram-negative bacteria and mediates the transport of oligopeptides across the inner membrane using ATP hydrolysis. This is part of the cell–cell communication using small peptides, in a much more complex signaling system that is not fully understood yet. However, it appears that the Opp is functioning in a quorum-sensing way, detecting the density of the bacterial population and possibly playing a role in biofilm regulation, and it may influence the expression of virulence factors by affecting intracellular pathways in a 3′,5′-cyclic diguanylate (c-di-GMP) signal [70,71,72,73].

The morphological integrity and the functionality of the flagellum influence the virulence of *V. alginolyticus* [12]. One of the regulating genes of the flagellum is *luxS*. The gene is also regulating the extracellular polysaccharide (EPS); it is involved in quorum sensing and influences biofilm formation [12,74,75,76].

Given the genomic detection of resistance determinants and a complex virulence profile, these findings underscore the need to integrate molecular diagnostics into routine workflows for otitis externa, particularly in immunocompromised patients, while also reinforcing the importance of genomic surveillance and antimicrobial stewardship to identify and manage emerging, atypical pathogens.

## 5. Conclusions

*Vibrio alginolyticus*, though a rare human pathogen, can act as a causative agent of otitis externa, particularly in immunocompromised individuals. The pathophysiology of such infections is influenced not only by host factors—such as diminished local immune responses—but also by the presence of specific virulence determinants. In this context, comprehensive genomic characterization has the potential to transform a routine ear discharge culture into an actionable diagnosis, guiding both targeted treatment and infection control strategies. This study highlights the value of integrating modern molecular tools, such as whole genome sequencing, with conventional diagnostic methods to confirm species identity, detect resistance genes, and assess virulence potential. Such an approach enables clinicians to distinguish true pathogens from colonizers, ultimately improving the clinical management of atypical otitis externa and other rare infections.

## Figures and Tables

**Table 1:** Mutations detected using SNP analysis.

Isolate	Gene	Mutation
Isolate A	*gyrB* *gyrB* *parE*	c.2024A>G c.2065C>G c.623G>A
Isolate B	*gyrB* *gyrB* *gyrA* *parE*	c.2024A>G c.2065C>G c.1870C>T c.623G>A

**Table 2:** Virulence factors obtained using the PeGAS pipeline.

Gene	Product	Isolate	% Identity	% Coverage	Start	End	Strand	Species Name
*tlh*	thermolabile hemolysin TLH thermolabile hemolysin (TLH)/lecithin-dependent hemolysin (LDH)	A	85.28	100.00	345,281	346,537	+	Vibrio species
		B	85.28	99.92	185,933	187,188	+	
*tyeA*	type III secretion system regulatory protein	A	81.40	100.00	102,289	102,573	+	Vibrio species
		B	81.40	100.00	1,354,811	1,355,095	+	
*vcrH*	type III secretion system chaperone VcrH	A	91.22	100.00	108,370	108,859	+	Vibrio species
		B	91.02	100.00	1,360,881	1,361,370	+	
*vopB*	type III secretion system translocator protein VopB	A	84.75	100.00	108,863	110,062	+	Vibrio species
		B	84.58	99.83	1,361,374	1,362,571	+	
*vopD*	type III secretion system translocator protein VopD	A	80.92	99.90	110,073	111,077	+	Vibrio species
		B	80.81	99.80	1,362,582	1,363,585	+	
*vopR*	type III secretion system effector VopR phosphoinositide-binding protein	A	80.86	99.90	89,423	90,399	+	Vibrio species
		B	80.86	99.80	1,341,958	1,342,933	+	
*vscF*	type III secretion system needle protein VscF	A	82.33	100.00	83,309	83,557	+	Vibrio species
		B	82.33	100.00	1,335,850	1,336,098	+	
*vscI*	type III secretion system inner rod protein VscI	A	83.14	99.71	84,561	84,904	+	Vibrio species
		B	83.14	99.42	1,337,102	1,337,444	+	
*vscN*	type III secretion system ATPase VscN	A	80.66	99.24	99,917	101,229	-	Vibrio species
		B	80.52	99.17	1,352,442	1,353,754	-	
*vscR*	type III secretion system C-ring protein VscR	A	83.82	99.69	96,618	97,266	-	Vibrio species
		B	83.67	99.54	1,349,149	1,349,796	-	
*vscS*	type III secretion system C-ring protein VscS	A	80.90	100.00	96,336	96,602	-	Vibrio species
		B	80.90	100.00	1,348,867	1,349,133	-	

**Table 3:** NCBI BLAST alignment of the two isolates with specific genes from the NIH GenBank ^®^. The GenBank^®^ links are found in the Supplementary Material section.

Isolate	Gene	Gene Description	GenBank^®^ ID	Genome Match Isolate A	Genome Match Isolate B
Hemolysin					
A, B	*hlyA*	hemolysin A	UAVI01000001.1	95.74%	99.49%
Colonization					
A, B	*toxR*	cholera toxin transcriptional activator	KJ579443.1	99.00%	99.55%
A, B	*collagenase*	collagenase	KX099763.1	98.13%	98.12%
B	*ompW*	major outer membrane protein	AY944132.1	-	98.60%
Biofilm					
A, B	*rpoN*	RNA polymerase sigma factor N	AB006709.1	94.58%	94.54%
A, B	*relA*	GTP diphosphokinase	75166643	98.96%	99.01%
A, B	*spoT*	bifunctional GTP diphosphokinase/guanosine-3′,5′-bis pyrophosphate 3′-pyrophosphohydrolase	69650475	99.15%	99.01%
A, B	*opp*	oligopeptide permease	AY566268.1	80.90%	80.85%
Motility—flagellum					
A, B	*luxS*	S-ribosylhomocysteine lyase	AY391122.1	99.81%	99.81%
Virulence proteins					
B	*vacB*	chitinase	AJ292004.1	-	90.51%
A	*proA*	gamma-glutamyl phosphate reductase	BATK01000019	99.44%	-
Antibiotic resistance genes					
A, B	*tet(34)*	oxytetracycline resistance determinant tet(34)	AB061440.1	82.31%	82.10%

## Data Availability

The data presented in this study are available on request from the corresponding author.

## Supplementary Materials

The following supporting information can be downloaded at: https://www.mdpi.com/article/10.3390/idr17050114/s1.
