# Supplementary material for: Uncommon Pathogens in Common Presentations: Genetic Profiling and Virulence Determinants of Vibrio alginolyticus Isolated from a Case of External Otitis
Source: Infect Dis Rep. 2025 Sep 12;17(5):114. doi: 10.3390/idr17050114 (PMC12452408; doi:10.3390/idr17050114)
Supplement: Supplementary file 1 [file idr-17-00114-s001.zip › idr-3821807-supplementary materials.pdf]

Gene	Gene description	GenBank® ID	Nucleotide link
hlyA	Hemolysin A	UAVI01000001.1	https://www.ncbi.nlm.nih.gov/nuccore/UAVI01000001.1
toxR	cholera toxin transcriptional activator	KJ579443.1	https://www.ncbi.nlm.nih.gov/nuccore/KJ579443.1
collagenase	collagenase	KX099763.1	https://www.ncbi.nlm.nih.gov/nuccore/KX099763.1
ompW	major outer membrane protein	AY944132.1	https://www.ncbi.nlm.nih.gov/nuccore/AY944132.1
rpoN	RNA polymerase sigma factor N	AB006709.1	https://www.ncbi.nlm.nih.gov/nuccore/AB006709.1
relA	GTP diphosphokinase	75166643	https://www.ncbi.nlm.nih.gov/gene/75166643
spoT	bifunctional GTP diphosphokinase/guanosine-3',5'-bis pyrophosphate 3'-pyrophosphohydrolase	69650475	https://www.ncbi.nlm.nih.gov/nuccore/BATK01000005.1
opp	Oligopeptide permease	AY566268.1	https://www.ncbi.nlm.nih.gov/nuccore/AY566268
luxS	S-ribosylhomocysteine lyase	AY391122.1	https://www.ncbi.nlm.nih.gov/nuccore/AY391122.1
vacB	Chitinase	AJ292004.1	https://www.ncbi.nlm.nih.gov/nuccore/AJ292004.1
proA	Gamma-glutamyl phosphate reductase	BATK01000019	https://www.ncbi.nlm.nih.gov/nuccore/BATK01000019.1
tet(34)	Oxytetracycline Resistance Determinant tet(34)	AB061440.1	https://www.ncbi.nlm.nih.gov/nuccore/AB061440.1
